# How MicroRNAs Command the Battle against Cancer

**DOI:** 10.3390/ijms25115865

**Published:** 2024-05-28

**Authors:** Hong Helena Wu, Sarah Leng, Consolato Sergi, Roger Leng

**Affiliations:** 1 370 Heritage Medical Research Center, Department of Laboratory Medicine and Pathology, University of Alberta, Edmonton, AB T6G 2S2, Canada; hwualberta@gmail.com; 2 Department of Laboratory Medicine and Pathology (5B4. 09), University of Alberta, Edmonton, AB T6G 2B7, Canadacsergi@cheo.on.ca (C.S.); 3 Division of Anatomical Pathology, Children’s Hospital of Eastern Ontario (CHEO), University of Ottawa, 401 Smyth Road, Ottawa, ON K1H 8L1, Canada

**Keywords:** miRNA, p53, p63, PTEN, therapy

## Abstract

MicroRNAs (miRNAs) are small RNA molecules that regulate more than 30% of genes in humans. Recent studies have revealed that miRNAs play a crucial role in tumorigenesis. Large sets of miRNAs in human tumors are under-expressed compared to normal tissues. Furthermore, experiments have shown that interference with miRNA processing enhances tumorigenesis. Multiple studies have documented the causal role of miRNAs in cancer, and miRNA-based anticancer therapies are currently being developed. This review primarily focuses on two key points: (1) miRNAs and their role in human cancer and (2) the regulation of tumor suppressors by miRNAs. The review discusses (a) the regulation of the tumor suppressor p53 by miRNA, (b) the critical role of the miR-144/451 cluster in regulating the Itch-p63-Ago2 pathway, and (c) the regulation of PTEN by miRNAs. Future research and the perspectives of miRNA in cancer are also discussed. Understanding these pathways will open avenues for therapeutic interventions targeting miRNA regulation.

## 1. miRNAs

MicroRNAs (miRNAs) are noncoding RNA molecules that regulate gene expression at transcription and translation levels [1,2,3,4]. They are 19–25 nucleotides (nt) long, with an average length of 22. The first miRNA, lin-4, was discovered in *C. elegans* by V. Ambros [5]. It was initially called stRNA (short temporal RNA) due to its restricted expression and its role in controlling the transition from larval to adult stage gene expression in *C. elegans*. The second miRNA, let-7, was subsequently identified in the development timing of *C. elegans* [6]. The homologues of the let-7 gene were then found in other animals, including humans [7]. The conservation of let-7 across species suggests a fundamental biological role for this small RNA. Humans have more than 1000 miRNAs that can target more than 30% of mammalian genes and are abundant in many types of human cells [8,9,10]. This makes miRNAs one of the most abundant classes of regulatory genes in humans. miRNAs are perceived as a key layer of post-transcriptional control within gene regulation. These miRNAs are highly conserved across species and have the common function of regulating gene expression at the post-transcriptional level [8,9,10].

## 2. miRNAs and Cancer

Numerous miRNAs have been found to have links to certain types of cancer [11,12]. Three observations made during the early stages of miRNA research suggested that they may play a role in human cancer. First, the first miRNAs discovered in roundworms *C. elegans* and fruit flies *Drosophila* were found to control cell growth and cell death, which can contribute to cancer if they are deregulated [5,13]. Second, when human miRNAs were discovered, many of them were found to be located in fragile sites in the genome or regions that are commonly amplified or deleted in human cancer [14]. Finally, miRNA expression was deregulated in malignant tumors and tumor cell lines compared to normal tissue [15,16,17]. It is still unknown whether altered miRNA expression is a cause or a consequence of malignant transformation.

## 3. miRNAs Function as an Oncogene or Tumor Suppressor

miRNAs are a type of RNA that plays a crucial role in the regulation of gene expression. They are short, non-coding RNAs that control target genes post-transcriptionally and have emerged as master regulators in various physiological and pathological processes, including oncogenesis [10,18,19,20,21,22]. Recent studies have shown that miRNAs regulate cell growth and apoptosis, making them potential targets for cancer therapies [19,22]. One of the first miRNAs discovered as an oncogene is miR-155, which is highly expressed in different types of cancer, including pediatric Burkitt lymphoma [21], Hodgkin disease [23], acute myeloid leukemia (AML) [24], lung cancer [25,26], and breast cancer [25,27]. However, the regulation of miR-155 and the mechanism of its overexpression in cancer are still unknown. Another oncogenic miRNA is miR-21, which is upregulated in various hematologic malignancies and solid tumors, including AML [24,28], glioblastoma [29], breast cancer [25], and liver tumors [30]. Understanding the role of miRNAs in cancer can help in the development of new diagnostic tools and therapies for cancer patients.

miRNAs can act as tumor suppressors by preventing the malignant transformation of a normal cell. In some cases, the expression of specific miRNAs is decreased in cancer cells, and these miRNAs are considered tumor suppressor genes [31]. For example, the miR15a/16-1 cluster is often downregulated or deleted in chronic lymphocytic leukemia (CLL) [32]. This cluster is inversely correlated with the expression of BCL-2, a key player in many types of human tumors [31]. This suggests that miRNAs may be involved in cancer formation by regulating cell growth and apoptosis. Ectopic expression of miR-16-1 has been shown to negatively regulate cell growth, induce apoptosis, and inhibit cell cycle progression in human cancer cell lines and in a leukemic xenograft model [33,34]. Similarly, the let-7 family of miRNAs is downregulated in many tumors, including lung and breast cancer [26,27,35], and overexpression of let-7 inhibited lung cancer cell growth in A549 lung adenocarcinoma cells [36]. However, miR-17-5p is described as a dual-role tumor suppressor/oncogene, which means that it can act according to the tissue and its transcriptome, including miRNA targets expressed in that particular tissue [25].

## 4. miRNAs and Cancer Therapy

miRNAs hold great promise as therapeutic agents because they regulate gene expression and influence cellular pathways involved in various diseases, including cancer, cardiovascular disorders, and neurological conditions [37,38,39,40,41,42,43,44,45,46,47,48]. The therapeutic potential of miRNAs stems from their ability to target multiple genes simultaneously, allowing for the modulation of complex biological processes. In this review, we focus on miRNAs and cancer therapy. (1) miRNA replacement therapy: In cancer, where miRNAs often act as tumor suppressors or oncogenes, restoring the expression of tumor suppressor miRNAs or inhibiting oncogenic miRNAs can be a therapeutic strategy. Synthetic miRNA mimics or viral vectors carrying miRNA sequences can be delivered to tumor cells to restore miRNA function and suppress tumor growth [41,42]. (2) miRNA inhibition therapy: In contrast, inhibition of oncogenic miRNAs using antisense oligonucleotides, antagomiRs, or miR-decoy can be effective in blocking their activity and inhibiting cancer progression [40,41]. (3) Drug delivery: Efficient delivery of miRNAs to target tissues remains a challenge for miRNA-based therapy. Various delivery systems, including lipid nanoparticles, viral vectors, and exosomes, are being developed to improve target cells’ stability, specificity, and uptake of miRNA [49,50]. (4) Combination therapy: miRNA-based therapies can be combined with conventional treatments such as chemotherapy, radiation therapy, or targeted therapies to improve treatment efficacy and overcome drug resistance. Combinatorial approaches can use synergistic interactions between miRNAs and other therapeutic modalities to improve patient outcomes [51,52,53].

Although miRNA-based therapy holds significant promise, several challenges need to be addressed, including optimization of delivery methods, minimizing off-target effects, and ensuring long-term safety and efficacy. However, ongoing research efforts continue to advance our understanding of miRNA biology and therapeutic applications, paving the way for the development of innovative treatments for a wide range of diseases. By expanding our discussion to include these additional miRNAs and their targets, we aim to provide a more comprehensive perspective on how miRNAs command the battle against cancer. This comprehensive approach underscores the potential as biomarkers and therapeutic agents, offering promising avenues for future cancer research and treatment.

## 5. Tumor Suppressor p53 and Tumors

**Figure 1:**
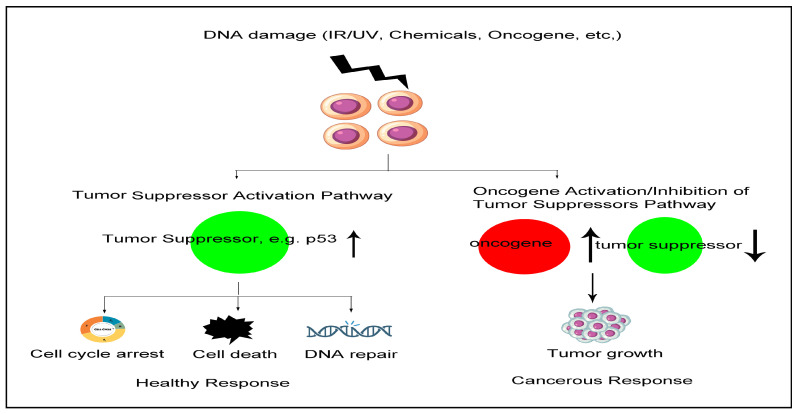
Response to DNA damage in cancer progression. This figure emphasizes the critical balance between tumor suppressor activation and oncogene activation in determining cellular fate following DNA damage. The proper function of tumor suppressors is essential for preventing cancer, while their inhibition or the activation of oncogenes can lead to malignant transformation.

The tumor suppressor p53 is inactivated in more than 50% of human tumors, and the p53 mutation is the most commonly observed genetic event in cancer cells [54,55,56,57]. Studies have demonstrated the crucial role of p53 in protecting genomes. Mice lacking p53 are highly susceptible to tumors [58], and germline mutations in p53 cause Li–Fraumeni syndrome, making individuals more prone to breast, brain, and adrenal gland cancers, as well as leukemias and sarcomas of bone and connective tissues [55,59]. The *p53* gene controls the cell cycle, initiates apoptotic cell death, and regulates DNA repair [56,60,61,62,63]. Mutations in p53 have been associated with more aggressive diseases and worse overall survival [64,65,66,67]. Inactivation of p53 has been associated with a poor prognosis and drug resistance in malignant tumors [55,57,68], indicating its importance in cancer pathogenesis. The regulation of p53 expression and stability is crucial in maintaining normal cell growth. Many upstream regulators and downstream effectors of p53 are believed to play a role in carcinogenesis in humans and other species. The response to DNA damage in cancer progression is summarized in Figure 1. miRNAs have also been shown to target p53 and/or components of p53 regulatory pathways, thereby directly and/or indirectly affecting its activities [69,70,71,72,73].

### 5.1. Regulation of p53 by miRNAs

Numerous miRNAs have been identified as regulators of p53, influencing its expression, activity, and downstream signaling pathways [69,74,75,76,77,78]. One of the well-studied miRNAs targeting p53 is miR-34a [79,80,81,82,83,84,85]. It is directly transactivated by p53 and forms a feedback loop that regulates the p53 function. miR-34a suppresses tumors by targeting multiple components of the p53 pathway, including SIRT1, which deacetylates and inhibits p53, and various anti-apoptotic and cell cycle regulatory genes [86]. Through its regulation of p53 signaling and downstream effectors, miR-34a maintains genomic stability and suppresses tumorigenesis. Several miRNAs indirectly regulate p53 expression or activity by targeting key regulators of the p53 pathway. For example, miR-125b and miR-504 target p53 inhibitors such as MDM2, increasing the stability and activity [87]. miR-192/215 and miR-504 can directly target the 3’UTR of p53 mRNA, leading to reduced expression of p53 [87,88]. miR-125a and miR-504 target p53 cofactors, such as the p300/CBP-associated factor (PCAF), which affects p53 acetylation and transcriptional activity [89]. Dysregulation of miRNA-mediated p53 regulation is involved in various diseases, particularly cancer [90]. Alterations in miRNA expression levels or mutations that affect miRNA binding sites in the p53 pathway can disrupt p53 function and contribute to tumorigenesis. Understanding the complex interplay between miRNAs and p53 signaling pathways can provide insights into developing novel therapeutic strategies for cancer treatment, such as miRNA-based therapies targeting the p53 pathway.

Our research has identified a microRNA called miR-1301, which plays an important role in the activity and function of p53 by targeting UBE4B [76]. It is worth noting that when miR-1301 is overexpressed, it inhibits the dissemination and metastasis of tumor cells in a p53-dependent manner. Our findings suggest that miR-1301 is a tumor suppressor that inhibits tumor cell migration and invasion by regulating the UBE4B-p53 pathway [76]. As p53 is a critical tumor suppressor, its regulation by miRNAs is a complex and actively researched area. Table 1 highlights the complex interaction between miRNAs and the p53 pathway, reflecting the nuanced regulatory mechanisms that govern cellular responses to stress and damage, impacting cancer development and progression. Three important insights are summarized below: (1). Upregulators of p53: The miRNAs that upregulate p53 generally do not directly target p53 but instead target negative regulators of p53 or elements involved in the cell cycle and proliferation. However, miR-214 is an exception that directly targets PSMD10 and ASF1B, which affect p53 function. (2). Downregulators of p53: Certain miRNAs downregulate p53 by directly binding to its 3’UTR. Some examples of these miRNAs are miR-25, miR-30d, miR-504, miR-380-5p, miR-92, and miR-141. Furthermore, miR-125b targets not only p53, but also a broader range of components within the p53 pro-apoptotic network. (3). Contextual functions: The effects of these miRNAs on p53 can vary depending on the context, such as the specific cell type or the state of the disease. For example, miR-125b has been implicated in the regulation of apoptosis and proliferation, impacting the conserved p53 network across vertebrates.

### 5.2. p63 Regulation by miRNAs

p63 is a homolog of the tumor suppressor p53 and can induce apoptosis and cell cycle arrest [119,120,121,122,123,124]. Although p63 has apoptotic activity, it rarely mutates in human tumors [125,126]. p63 deficiency is embryonically lethal in mice, causing severe developmental abnormalities, including a lack of epithelium and abnormalities of limb development [127,128]. However, recent studies have found that p63 expression is lost or reduced in certain human tumors. This suggests that p63 can potentially suppress tumors [129,130,131,132]. Flores et al. (2005) have shown that loss of p63 can cooperate with loss of p53 in tumor suppression [129]. Furthermore, mutations in both p63 and p53 can lead to more aggressive tumor growth [133,134]. Metastatic tumors that progress faster tend to have lower levels of p63 expression, suggesting that loss of p63 accelerates tumorigenesis and metastatic spread [135].

Less is known about the regulation of p63 by miRNAs compared to p53. However, emerging evidence suggests that miRNAs may play an important role in modulating p63 expression and activity in various cellular processes, including development, differentiation, and cancer. Several miRNAs, such as miR-203, miR-130b, miR-205, and miR-944, have been implicated in regulating p63 expression or activity [136,137,138,139,140,141,142]. These miRNAs can target different isoforms of p63 and modulate its transcriptional activity, protein stability, or subcellular localization.

We have discovered that the expression of miR-144/451 leads to an increase in p63 protein levels [143]. Furthermore, TAp63 transactivates the miR-144/451 cluster promoter, resulting in a positive feedback loop. miR-144 triggers apoptosis and reduces cell invasion in a TAp63-dependent manner. We found that Itch, an E3 ubiquitin ligase for p63, is directly targeted by miR-144. We also observed that the association between Ago2 and miR-451 is dependent on TAp63, implying the critical role of TAp63 in the biogenesis of miR-451 mediated by Ago2 [143]. This report highlights that miR-144 is a crucial regulator of the Itch-p63-Ago2 pathway, which could have implications for developing novel therapies for treating human tumors. Table 2 summarizes the p63 regulation by miRNAs. The table represents the currently known interactions, but further research is likely to identify additional miRNAs. The impact of miRNAs on p63 can vary based on cell type, tissue context, and specific isoforms of p63 involved (TAp63 vs. ΔNp63). The regulation of p63 by miRNAs underscores its critical role in epithelial cell fate, tumorigenesis, and cellular stress responses.

### 5.3. Regulation of PTEN by miRNAs

The phosphatase and tensin homolog (PTEN) is a well-known tumor suppressor involved in the PI3K/AKT signaling pathway. PTEN’s loss or downregulation is frequently observed in various cancers. Regulation of PTEN by miRNAs is a crucial area of research. Multiple miRNAs have been identified to target PTEN directly, leading to decreased expression and tumor progression. Understanding the interplay between these miRNAs and PTEN offers potential therapeutic avenues to restore PTEN function in cancer cells [153,154].

Among the miRNAs involved in PTEN regulation, miR-21 stands out as particularly significant due to its frequent upregulation in various human cancers and its direct targeting of PTEN. miR-21 overexpression has been shown to promote the growth and metastasis of specific cancers, including non-small cell lung cancer, colorectal carcinoma, ovarian cancer, and triple-negative breast cancers [30,153,155,156,157,158,159]. Furthermore, miR-21 and miR-181b-1 have been implicated in inhibiting PTEN and cylindromatosis (CYLD) tumor suppressor functions, leading to increased NF-kB activity and linking inflammation to cancer [160]. This evidence highlights the pivotal role of miR-21 in the oncogenic process through the modulation of PTEN expression, underlining its importance among miRNAs involved in PTEN regulation.

Other miRNAs, such as miR-214, miR-93, and miR-130a, also modulate PTEN expression and are associated with resistance to therapies such as cisplatin in ovarian cancer cells [161,162,163]. Furthermore, miR-221 and miR-222 have been associated with aggressive forms of cancer, such as non-small cell lung cancer (NSCLC), hepatocarcinoma, and breast cancer, targeting PTEN and contributing to resistance to treatment [164,165].

The intricate regulation of PTEN by miRNAs is further complicated by the presence of competing endogenous RNAs (ceRNAs), which can sequester miRNAs, thus modulating the levels of PTEN. For example, the PTEN pseudogene (PTENpg1) can act as a ceRNA, sequestering miRNAs away from PTEN mRNA and, therefore, enhancing its expression [166,167].

**Figure 2:**
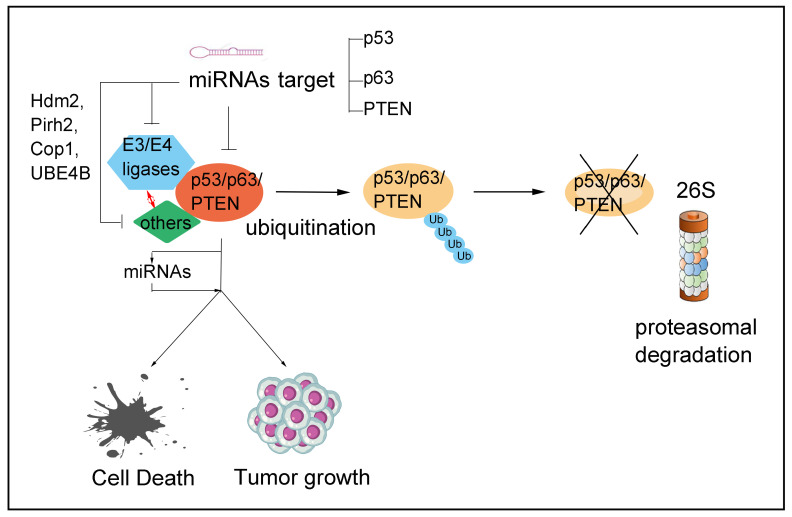
Illustrates how miRNAs regulate tumor suppressors’ expression or activity. They can also affect their stability and activity by targeting an E3 ligase, thus influencing cell proliferation and cancer progression. Moreover, miRNAs can promote cell death. The figure provides a summary of tumor suppressors that are regulated by miRNAs.

We identified that miR-498 directly targeted the 3’UTR of PTEN mRNA and reduced PTEN protein levels in triple-negative breast cancer (TNBC) cells [168]. miR-498 promoted cell proliferation and cell cycle progression in TNBC cells in a PTEN-dependent manner. Suppressing miR-498 expression affected the oncogenic effects of miR-498 on cell proliferation and migration. These results provide new information on the downregulation of PTEN and indicate a potential therapeutic target for the treatment of TNBC [168]. Future directions in the regulation of PTEN by miRNAs include a deeper exploration of the complex network of miRNA-PTEN interactions and how they affect cancer progression and resistance to treatments. The challenges lie in the vast diversity of miRNAs and their targets and the context-dependent effects of miRNAs, which can vary between different types of tissues and stages of disease. Understanding these dynamics is crucial for developing miRNA-based therapeutic strategies that could potentially target PTEN regulation to combat cancer more effectively. Table 3 highlights the essential role of miRNAs in the regulation of PTEN expression, which influences cancer progression through various mechanisms. Several factors need to be considered when studying the interaction between miRNAs and PTEN. First, the relationship between them is complex and context-dependent, which means that it can vary depending on the type of cancer, the cellular environment, and the specific expression levels of miRNA. Second, some miRNAs may not directly target PTEN, but can still affect its expression through upstream signaling pathways or other regulatory molecules. Finally, studying the role of miRNAs in PTEN regulation can lead to developing new therapeutic strategies for cancers involving PTEN dysfunction. We propose a model to illustrate how miRNAs regulate tumor suppressors’ expression or activity (Figure 2).

## 6. Diagnostic, Prognostic, and Predictive Potential of miRNAs in Cancer

### 6.1. Diagnostic Potential of miRNAs

#### 6.1.1. Early Detection of Cancers

miRNAs such as miR-34a, miR-125b, and miR-504, which are involved in p53 regulation [84,87,108], miR-203, miR-130b, and miR-944, which influence p63 expression or activity [142,145,148], and miRNAs such as miR-21 and miR-498 that directly target PTEN are frequently upregulated in various cancers such as triple-negative breast cancer and colorectal carcinoma [155,168]. Altered levels of these miRNAs might be detectable in bodily fluids before traditional clinical symptoms manifest, offering a non-invasive diagnostic tool.

#### 6.1.2. Cancer Typing

miRNA profiles that regulate p63 may help differentiate cancer subtypes, particularly epithelial cancers where p63 plays a critical role in development and differentiation. Measuring specific miRNAs could help distinguish between cancer types and even subtypes, informing treatment strategies.

### 6.2. Prognostic Potential of miRNAs

#### 6.2.1. Survival and Treatment Outcomes

miRNAs such as miR-34a and miR-1301, which regulate key components of the p53 pathway, have been linked to tumor suppression and genomic stability [76,84]. Their expression levels could potentially predict patient survival, likelihood of recurrence, and response to therapy. Higher levels of tumor suppressor miRNAs might correlate with better prognosis. miRNAs that modulate p63, such as miR-144 and miR-451, might be associated with better or worse outcomes based on their impact on p63 expression levels and tumor suppressor functions [143]. The role in triggering apoptosis and reducing cell invasion could be correlated with a favorable prognosis in cancers where p63 is active. miRNAs that downregulate PTEN, such as miR-21 and miR-221/222, have been linked with poor prognosis and more aggressive cancer phenotypes [155,164,165]. Patients exhibiting high levels of these miRNAs may have a worse prognosis, with faster disease progression and lower overall survival rates.

#### 6.2.2. Assessing Disease Aggression

miRNAs that downregulate p53, such as miR-504 and miR-380-5p, could serve as biomarkers for more aggressive cancers [87,109]. Their presence and levels could inform clinicians about the likely disease course and aggressiveness, helping to tailor more aggressive treatment plans where necessary. Lower levels of p63 have been associated with more aggressive and metastatic tumor behaviors. miRNAs that decrease p63 expression or destabilize its protein could serve as indicators of more aggressive disease courses, aiding in predicting the likelihood of metastasis and overall disease aggressiveness. miRNAs such as miR-21 that promote cancer growth and metastasis by suppressing PTEN can also serve as markers to gauge disease progression [155,156]. Higher expression of such miRNAs could indicate a higher likelihood of metastatic spread and more aggressive disease, informing treatment intensity and monitoring strategies.

### 6.3. Predictive Potential of miRNAs

#### 6.3.1. Prediction of Treatment Response

miRNAs such as miR-34a and miR-1301, targeting elements of the p53 pathway, could predict how well a patient might respond to specific therapies, particularly those that aim to activate p53. For example, patients with high expression of miR-34a might respond favorably to therapies that rely on intact p53 signaling. Since miRNAs such as miR-144 directly target components such as Itch, which are involved in the regulation of p63, their expression profiles could predict how a tumor might respond to therapies aiming to modulate the p63 pathway [143]. For example, tumors with high levels of miR-144 might respond better to therapies that aim to enhance p63’s tumor suppressive activities. miRNAs such as miR-214, miR-93, and miR-130a, which modulate PTEN expression, have been associated with resistance to therapies like cisplatin in ovarian cancer [161,162,163]. Profiling these miRNAs in patients could predict their response to specific treatments, enabling personalized therapy plans that could bypass or counteract resistance mechanisms.

#### 6.3.2. Personalized Medicine Applications

Understanding the specific interactions between miRNAs and p53 can help in customizing therapeutic approaches. For example, if a patient has downregulated miR-1301, therapies designed to enhance its expression could be more effective in restoring normal p53 function and potentially inhibiting tumor growth and spread. Understanding the specific miRNA-p63 interactions could guide the development of targeted therapies, such as miRNA mimics or antagomiRs, to either upregulate or downregulate p63 activity in cancer treatments, depending on the desired therapeutic effect. Identifying the presence of specific miRNAs that target PTEN can guide the development and use of targeted therapies, such as miRNA mimics or inhibitors. For example, in cancers where miR-498 is found to downregulate PTEN, therapies aimed at inhibiting miR-498 could be effective.

## 7. Tissue-Specific Roles of miRNAs in Cancer

The tissue-specific expression and activity of miRNAs are crucial for understanding their roles in the pathogenesis and progression of various cancers.

### 7.1. Tissue-Specific Expression Patterns

The expression of specific miRNAs can be inherently tied to particular tissues. For example, miR-21 is upregulated in lung and breast tissues in cancerous states, distinctly influencing tumor progression in each tissue type.

### 7.2. Differential Roles in Cancer

miR-34a acts differently in colorectal cancer compared to its role in prostate cancer, according to its interaction with tissue-specific cofactors and targets [193].

### 7.3. Impact on Metastasis and Tissue Invasion

For example, miR-10b promotes metastasis in breast cancer but plays a different role in pancreatic cancer [194,195].

### 7.4. Clinical Implications

For example, miR-205 is a potent marker for skin and breast cancers, but plays a variety of roles in these tissues, which could influence therapeutic strategies [196].

## 8. Role of miRNAs in Modulating Immune cell Dynamics in the Cancer Microenvironment

### 8.1. miRNAs and Immune Cell Recruitment

miR-21 has been shown to reduce the expression of PDCD4, a pro-inflammatory protein, potentially diminishing the antitumor immune response to tumors and facilitating tumor growth [197].

### 8.2. Impact on Immune Evasion

miR-221 and miR-222 target PTEN and also influence immune surveillance by affecting the expression of immune checkpoint molecules such as PD-L1 [198]. This mechanism can lead to increased immune evasion, particularly in cancers where PTEN is already compromised.

### 8.3. Regulation of Immune-Related Signaling Pathways

miR-34a, directly transactivated by p53, influences tumor cells and impacts immune cells by modulating cytokine profiles and T-cell activation, critical for immune surveillance and antitumor immunity [193,199].

### 8.4. Interaction between p63, miRNAs, and Immune Cells

miR-203 and miR-205 regulate p63 and influence epithelial-to-mesenchymal transition (EMT) [200,201]. EMT is crucial for metastasis and for the creation of an immunosuppressive microenvironment that limits the efficacy of immune cells.

### 8.5. Therapeutic Implications

Understanding these interactions opens new avenues for therapies targeting these miRNAs, which could enhance immune response while controlling tumor growth. For example, inhibiting miR-21 or miR-221/222 could restore PTEN function and enhance immune cell activity within the tumor microenvironment.

## 9. Role of miRNAs in Tumor Differentiation, Evolution, and Metastasis

### 9.1. miRNAs and Tumor Differentiation

#### 9.1.1. Influence on Cell Fate

Some members of the miR-200 family influence the epithelial–mesenchymal transition (EMT), a key process in tumor cell differentiation and metastasis.

#### 9.1.2. Regulation of Differentiation Markers

miRNAs modulate the expression of markers that are crucial for maintaining stemness or differentiation within tumor cells, such as the role in regulating CD44, a cancer stem cell marker [202].

### 9.2. miRNAs and Tumor Evolution

#### 9.2.1. Genetic and Epigenetic Modulation

miRNAs contribute to the genetic and epigenetic changes within tumors, affecting tumor heterogeneity and evolution; for example, miR-21’s role in promoting oncogenic mutations by targeting multiple tumor suppressor genes, including PTEN.

#### 9.2.2. Adaptation to the Microenvironment

miR-155 influences the ability of tumor cells to adapt to and manipulate their microenvironment, facilitating survival and growth under various stress conditions.

### 9.3. miRNAs and Metastases

#### 9.3.1. Promotion of the Metastatic Cascade

miRNAs such as miR-10b and miR-21 facilitate different stages of the metastatic cascade, from local invasion, intravasation, survival in circulation, and extravasation, to colonization at distant sites.

#### 9.3.2. Interaction with the Metastatic Niche

miRNAs prepare distant organs for tumor cell colonization (premetastatic niche formation), including the impact on metastatic liver colonization in breast cancer.

## 10. Conclusions and Perspectives

miRNAs play critical roles in regulating tumor suppressor(s) or oncogene(s) expression, activity, and downstream signaling pathways. Through direct and indirect mechanisms, miRNAs can influence various aspects of tumor suppressor/or oncogene function, maintaining cell homeostasis and suppressing tumorigenesis. Future research efforts may focus on unraveling the functional significance of miRNA-mediated tumor suppressor/oncogene regulation in normal physiological processes and disease contexts, such as cancer and developmental disorders. Furthermore, elucidating the cross-talk between miRNA-regulated pathways and tumor suppressor/oncogene signaling networks could provide insight into complex regulatory mechanisms. Developing innovative approaches to targeted modulation of miRNA activity, such as miRNA mimics, inhibitors, or delivery systems, can offer new therapeutic opportunities for diseases associated with dysregulated p53/p63 signaling. Challenges in studying tumor suppressor/oncogene regulation by miRNAs include the complexity of the regulatory network, the context-dependent effects of miRNAs, and the need for robust experimental models and methodologies to validate miRNA–target interactions and functional outcomes. The challenges of studying the regulation of p63 by miRNAs include the diversity of p63 isoforms, the context-dependent functions of p63 in different cell types and tissues, and the need for sophisticated experimental approaches to dissect the molecular mechanisms underlying miRNA–p63 interactions. Future research efforts aimed at elucidating the complex interplay between miRNAs and tumor suppressor/oncogene signaling networks hold promise for uncovering new insights into cellular processes and disease mechanisms, with potential implications for developing novel therapeutic strategies.

## Figures and Tables

**Table 1:** p53 regulation by miRNAs: a more comprehensive view.

miRNAs	Function to p53	Regulation of p53	Cancer Type	Model	Type of Evidence	References
miR-122	Activates p53 by interacting with p53-regulating factors such as MDM2, SIRT1, YY1.	Upregulates	Hepatocellular carcinoma	In vitro cell line	qPCR, Western blot	[91,92]
miR-34	Directly regulated by p53, it represses several cell cycle and proliferation targets.	Upregulates	Multiple types	In vivo xenograft, in vitro cell line	qPCR, Western blot, RIP	[79,80,81,82,83,84,85]
miR-29	Activates p53 indirectly by targeting MDM2.	Upregulates	Lung cancer	In vitro cell line	qPCR, Western blot	[93,94]
miR-192	Activates p53 indirectly by targeting MDM2.	Upregulates	Colorectal cancer	In vitro cell line	qPCR, Western blot	[88,95]
miR-194	Works similarly to miR-192 in activating p53.	Upregulates	Multiple types	In vitro cell line	qPCR, Western blot	[69,89,96,97,98]
miR-215	Along with miR-192 and miR-194, MDM2 is targeted to activate p53.	Upregulates	Multiple types	In vitro cell line	qPCR, Western blot	[69,88,96,97,98]
miR-605	Activates p53 indirectly by targeting MDM2.	Upregulates	Lung cancer	In vitro cell line	Western blot	[69,96,97,98]
miR-214	Positively regulates p53 by suppressing PSMD10 and ASF1B, direct targets of miR-214.	Upregulates	Multiple types	In vitro cell line	qPCR, Western blot	[89,99]
miR-145	Activates p53 indirectly by targeting MDM2.	Upregulates	Multiple types	In vitro cell line	qPCR, Western blot	[100]
miR-1827	Activates p53 indirectly by targeting MDM2.	Upregulates	Multiple types	In vivo xenograft, in vitro cell line	qPCR, Western blot	[101]
miR-18b	Activates p53 indirectly by targeting MDM2.	Upregulates	melanoma	In vivo xenograft, in vitro cell line	qPCR, Western blot	[102]
miR-339-5p	Activates p53 indirectly by targeting MDM2.	Upregulates	Multiple types	In vitro cell line	qPCR, Western blot	[103]
miR-1301	Activates p53 indirectly by targeting UBE4B.	Upregulates	Multiple types	In vitro cell line	qPCR, Western blot	[76]
miR-449	Induces p53 activation by inhibiting HDAC1 and SIRT1.	Upregulates	Gastric cancer	In vitro cell line	qPCR, Western blot	[104]
miR-32	Activates p53 indirectly by targeting MDM2 and TSC1.	Upregulates	Glioblastoma	In vitro cell line	qPCR, Western blot	[105]
miR-542-3p	Activates p53 indirectly by targeting MDM2 and RPS23.	Upregulates	Neuroblastoma	In vitro cell line	qPCR, Western blot	[106]
miR-661	Activates p53 indirectly by targeting MDM2 and MDM4.	Upregulates	Breast cancer	In vitro cell line	qPCR, Western blot	[75]
miR-3928	Activates p53 indirectly, by targeting MDM2, CD44, DDX3X, HMGA2, etc.	Upregulates	Glioblastoma	In vivo xenograft, in vitro cell line	qPCR, Western blot	[107]
miR-25	Negatively regulates p53 by directly binding to its 3′UTR.	Downregulates	Multiple myeloma	In vitro cell line	qPCR, Western blot	[70]
miR-30d	Negatively regulates p53, similar to miR-25.	Downregulates	Multiple myeloma	In vitro cell line	qPCR, Western blot	[70]
miR-125b	Target p53 and several components of the p53 pro-apoptotic network, including BAK1 and PUMA.	Downregulates	Brain cancer	In vitro cell line, zebrafish	qPCR, Western blot	[108]
miR-504	Directly targets p53’s 3′UTR, negatively regulating it.	Downregulates	Multiple types	In vivo xenograft, in vitro cell line	qPCR, Western blot	[87]
miR-380-5p	Directly targets p53’s 3’UTR, acting as a negative regulator.	Downregulates	Brain cancer	In vivo xenograft, in vitro cell line	qPCR, Western blot	[109]
miR-92	Negatively regulates p53.	Downregulates	Prostate cancer	In vitro cell line	qPCR, Western blot	[110]
miR-141	Negatively affects p53 by binding to its 3′UTR.	Downregulates	Glioblastoma	In vitro cell line	qPCR, Western blot	[111]
miR-33	Directly targets p53’s 3′UTR.	Downregulates	Hepatocellular carcinoma	In vitro cell line	qPCR, Western blot	[112]
miR-19b	Directly targets p53’s 3′UTR.	Downregulates	Multiple types	In vivo xenograft, in vitro cell line	qPCR, Western blot	[113]
miR-98	Directly targets p53’s 3′UTR.	Downregulates	Lung cancer	In vitro cell line	qPCR, Western blot	[114]
miR-150-5p	Directly targets p53’s 3′UTR.	Downregulates	Colorectal cancer	In vitro cell line	qPCR, Western blot	[115]
miR-375	Directly targets p53’s 3′UTR.	Downregulates	Gastric cancer	In vitro cell line	qPCR, Western blot	[116]
miR-1285	Directly targets p53’s 3′UTR.	Downregulates	Multiple types	In vitro cell line	qPCR, Western blot	[117]
miR-3151	Directly targets p53’s 3′UTR.	Downregulates	Acute leukemia	In vitro cell line	qPCR, Western blot	[118]

Please note that this is not an exhaustive list, and numerous other miRNAs have been shown to regulate p53 expression.

**Table 2:** p63 regulation by miRNAs.

miRNA	Function to p63	Regulation of p63	Cancer Type	Model	Type of Evidence	References
miR-574-3p	Inhibited by iASPP, p63 expression is maintained due to inhibition of miR-574-3p, which targets p63.	Upregulates	Skin cancer	In vitro cell line	qPCR, Western blot	[144]
miR-720	Like miR-574-3p, its expression is inhibited by iASPP to maintain p63 expression.	Upregulates	Skin cancer	In vitro cell line	qPCR, Western blot	[144]
miR-144	Activates p63 indirectly by targeting Itch.	Upregulates	Breast cancer	In vitro cell line	qPCR, Western blot	[143]
miR-203	Represses p63 expression in supra-basal epithelial cells, contributing to the border between progenitor and differentiated epithelial cells.	Downregulates	Head and neck cancer	In vitro cell line	qPCR, Western blot	[145]
miR-92	Targets ΔNp63α and β, affecting expression in keratinocyte and myeloid cells, respectively.	Downregulates	Leukemia	In vitro cell line	qPCR, Western blot, RIP	[146]
miR-302	Suppresses p63 expression in germ cells.	Downregulates	Testicular cancer	In vitro cell line	qPCR, Western blot	[147]
miR-130b	Directly targets p63’s 3′UTR.	Downregulates	Multiple types	In vitro cell line	qPCR, Western blot	[148]
miR-181a	Directly targets p63’s 3′UTR.	Downregulates	Squamous carcinoma	In vitro cell line	qPCR, Western blot	[149]
miR-196a	Directly targets p63’s 3′UTR.	Downregulates	Breast cancer	In vitro cell line	qPCR, Western blot	[150]
miR-21	Directly targets p63’s 3′UTR.	Downregulates	Multiple types	In vivo xenograft, in vitro cell line	qPCR, Western blot	[151,152]
miR-30b/c	Directly targets p63’s 3′UTR.	Downregulates	Glioblastoma	In vitro cell line	qPCR, Western blot	[152]
miR-374a	Directly targets p63’s 3′UTR.	Downregulates	Squamous carcinoma	In vitro cell line	qPCR, Western blot	[149]
miR-519a	Directly targets p63’s 3′UTR.	Downregulates	Squamous carcinoma	In vitro cell line	qPCR, Western blot	[149]
miR-630	Directly targets p63’s 3′UTR.	Downregulates	Squamous carcinoma	In vitro cell line	qPCR, Western blot	[149]
miR-885-3p	Directly targets p63’s 3′UTR.	Downregulates	Squamous carcinoma	In vitro cell line	qPCR, Western blot	[149]
miR-138	Directly targets p63’s 3′UTR.	Downregulates	Multiple types	In vitro cell line	qPCR, Western blot	[148]
miR-944	Directly targets p63’s 3′UTR.	Downregulates	Cervical cancer	In vitro cell line	qPCR, Western blot	[142]

**Table 3:** Regulation of PTEN by miRNAs.

miRNAs	Function to PTEN	Regulation of PTEN	Cancer Type	Model	Type of Evidence	References
miR-21	Promotes cancer growth and metastasis by targeting PTEN.	Downregulates	Various, including lung and breast	In vitro cell line, in vivo xenograft	qPCR, Western blot	[30,153,155,156,157,158,159]
miR-214	Reduces PTEN expression, enhancing cell survival and drug resistance.	Downregulates	Nasopharyngeal carcinoma	In vitro cell line	qPCR, Western blot	[162]
miR-93	It is associated with resistance to cisplatin in ovarian cancer cells by targeting PTEN.	Downregulates	Breast cancer	In vitro cell line	qPCR, Western blot	[161]
miR-130a	Linked to cisplatin resistance by directly targeting PTEN,	Downregulates	Breast cancer	In vitro cell line	qPCR, Western blot	[163]
miR-498	Directly targeting the 3’UTR of PTEN.	Downregulates	Breast cancer (TNBC)	In vitro cell line	qPCR, Western blot	[168]
miR-221/222	Directly targeting the 3’UTR of PTEN.	Downregulates	Non-small cell lung cancer	In vitro cell line	qPCR, Western blot	[164,165]
miR-26a	Directly targeting the 3’UTR of PTEN.	Downregulates	Multiple types	In vitro cell line	qPCR, Western blot	[169,170]
miR-19-3p	Targets PTEN to regulate cervical cancer cell proliferation, invasion, and Autophagy.	Downregulates	Cervical cancer	In vitro cell line	qPCR, Western blot	[153,171]
miR-216a/217	promote metastasis by targeting PTEN.	Downregulates	Ovarian cancer	In vitro cell line	qPCR, Western blot	[172,173]
miR-22	Directly targeting the 3’UTR of PTEN.	Downregulates	Multiple types	In vitro cell line	qPCR, Western blot	[174,175]
miR-182-5p	Inhibits cell proliferation and Invasion of Breast Cancer Cells by targeting PTEN.	Downregulates	Breast cancer	In vitro cell line	qPCR, Western blot	[176]
miR-153	activated the AKT signaling and downregulated FOXO1 transcriptional activity. By targeting PTEN.	Downregulates	Prostate cancer	In vitro cell line	qPCR, Western blot	[177,178]
miR-106b	Directly targeting the 3’UTR of PTEN.	Downregulates	Breast cancer	In vitro cell line	qPCR, Western blot	[179,180]
miR-382-5p	Directly targeting the 3’UTR of PTEN.	Downregulates	Leukemia	In vitro cell line	qPCR, Western blot	[181]
miR-144/451	Promote cell proliferation by targeting PTEN.	Downregulates	Insulinomas	In vitro cell line	qPCR, Western blot	[182]
miR-29	promotes osteosarcoma cell proliferation and migration by targeting PTEN.	Downregulates	Osteosarcoma	In vitro cell line	qPCR, Western blot	[183]
miR-200a	Directly targeting the 3’UTR of PTEN.	Downregulates	Ovarian cancer	In vitro cell line	qPCR, Western blot	[184]
miR-552	Directly targeting the 3’UTR of PTEN.	Downregulates	Ovarian cancer	In vitro cell line	qPCR, Western blot	[185]
miR-186	Directly targeting the 3’UTR of PTEN.	Downregulates	Ovarian cancer	In vitro cell line	qPCR, Western blot	[186]
miR-371	Directly targeting the 3’UTR of PTEN.	Downregulates	Hepatocellular carcinoma	In vitro cell line	qPCR, Western blot	[187]
miR-155	Directly targeting the 3’UTR of PTEN.	Downregulates	Hepatocellular carcinoma	In vitro cell line	qPCR, Western blot	[188]
miR-106a	Directly targeting the 3’UTR of PTEN.	Downregulates	Prostate cancer	In vitro cell line	qPCR, Western blot	[189]
miR-183-5p	Directly targeting the 3’UTR of PTEN.	Downregulates	Non-small cell lung cancer	In vitro cell line	qPCR, Western blot	[190]
miR-486	Directly targeting the 3’UTR of PTEN.	Downregulates	Pancreatic cancer	In vitro cell line	qPCR, Western blot	[191]
miR-142-5p	Directly targeting the 3’UTR of PTEN.	Downregulates	Intrahepatic cholangiocarcinoma	In vitro cell line	qPCR, Western blot	[192]

## Data Availability

All other relevant data supporting the findings of this study are available in the article.
